# Differential diagnosis of relapsing polychondritis from asthma by 18‐fluoro‐2‐deoxyglucose positron emission tomography and computed tomography

**DOI:** 10.1002/ccr3.3933

**Published:** 2021-02-12

**Authors:** Mariko Ono, Yuki Maeda, Nobuyuki Koyama, Hiroyuki Nakamura, Kazutetsu Aoshiba

**Affiliations:** ^1^ Department of Respiratory Medicine Tokyo Medical University Ibaraki Medical Center Inashiki Japan; ^2^ Department of Respiratory Medicine Tokyo Medical University Shinjuku‐ku Japan; ^3^ Department of Clinical Oncology Tokyo Medical University Ibaraki Medical Center Inashiki Japan

**Keywords:** 18‐fluoro‐2‐deoxyglucose positron emission tomography and computed tomography, bronchial asthma, cough, relapsing polychondritis

## Abstract

About a half of all patients with relapsing polychondritis show airway involvement, which is a major cause of morbidity and mortality from this disease. FDG‐PET/CT is useful in the differential diagnosis of relapsing polychondritis from asthma.

## Graphical abstract

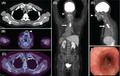

Clinicians should recognize relapsing polychondritis as a condition that must be differentiated from asthma unresponsive to inhaled corticosteroids and/or bronchodilators. Herein, we report a case of relapsing polychondritis in which FDG‐PET/CT was useful in the differential diagnosis of relapsing polychondritis from asthma.

A 67‐year‐old woman presented with a 2 weeks history of persistent cough. She had been diagnosed as having ulcerative colitis 27 years earlier and was taking mesalazine. She also had had an episode of bronchial asthma 20 years ago. Chest auscultation revealed no crackles or wheezes. The white cell count was 6800/μL, and the serum level of C‐reactive protein was 0.99 mg/dL. A plain chest X‐ray showed no abnormal findings. The patient was suspected as having a relapse of asthma and initiated treatment with an inhaled corticosteroid combined with a long‐acting β2 receptor agonist, which proved ineffective. Computed tomographic (CT) imaging of the chest revealed tracheal wall thickening predominantly of the anterior and lateral walls (Figure 1A). 18‐fluoro‐2‐deoxyglucose positron emission tomography and CT (FDG‐PET/CT), which was performed to exclude malignancy, showed increased FDG accumulation in the nasal alar cartilages and the anterior and lateral portions of trachea (Figure 1B‐E), suggesting the diagnosis of relapsing polychondritis. Fiberoptic bronchoscopy revealed mucosal edema and cartilaginous hypertrophy in the trachea (Figure 1F), which were consistent with relapsing polychondritis. The clinical symptoms improved with the start of oral prednisolone therapy (20 mg/day). In conclusion, FDG‐PET/CT was useful for the differential diagnosis of relapsing polychondritis from asthma.^1^

**FIGURE 1:**
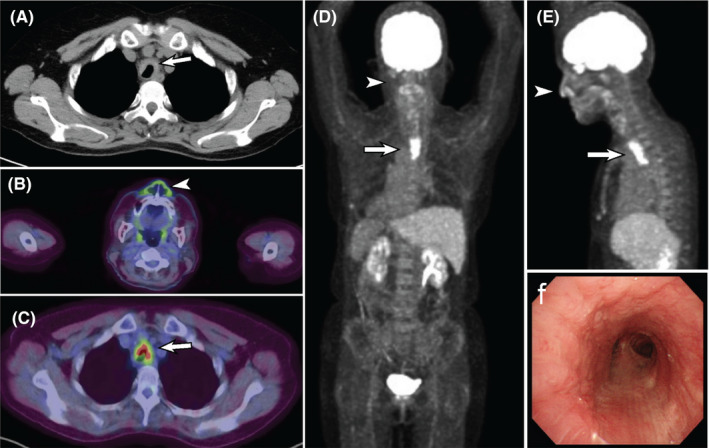
A, Plain CT image of the chest showing tracheal wall thickening, predominantly in the anterior and lateral walls (*arrow*). B‐E, Axial PET‐CT fusion images (B and C) and maximal intensity projection (MIP) images (D and E) showed increased FDG accumulation in the nasal alar cartilages (*arrowheads*) and trachea (*arrows*); the posterior membranous portion of the trachea appears to be uninvolved. F, Fiberoptic bronchoscopic image showing mucosal edema and cartilaginous hypertrophy of the trachea

## CONFLICT OF INTEREST

The authors have no conflicts of interest to declare.

## AUTHORS' CONTRIBUTION

MO and KA: wrote the first draft. YM, NK, and HN: contributed to the clinical management of the patient and revised the manuscript.

## ETHICAL APPROVAL

There are no specific ethics comments.

## INFORMED CONSENT

Informed consent from the patient was obtained for publication of the case report.

## Data Availability

The data that support the findings are available on request.
